# Update on Male Infertility

**DOI:** 10.3390/jcm10204771

**Published:** 2021-10-18

**Authors:** Erlisa Bardhi, Panagiotis Drakopoulos

**Affiliations:** 1 Centre for Reproductive Medicine, Universitair Ziekenhuis Brussel, Vrije Universiteit Brussel, Laarbeeklaan 101, 1090 Brussels, Belgium; erlibardhi@gmail.com; 2 Department of Obstetrics and Gynaecology, University of Alexandria, Alexandria 21526, Egypt

Infertility, defined as the failure to conceive after one year of regular intercourse without the use of contraception, in women less than 35 years of age remains a unique medical condition, as it involves a couple rather than a single individual. It can affect around 15% of couples, and it is believed that male factors contribute to the underlying or contributory causal factors in up to half of these cases, despite a paucity of global, high-quality data on the subject [1,2,3]. In this context, a detailed understanding of different etiologies and risk factors for male infertility is mandatory for optimal couple evaluation and treatment. Generally, causes of male infertility fall into four large categories, including primary testicular defects in spermatogenesis, systemic and/or endocrine disorders resulting in hypogonadotropic hypogonadism, sperm transport disorders and idiopathic male infertility [2,4,5]. Given the growing awareness on the subject, the impact it has on the psyche and wellbeing of men worldwide and the evidence of a decline in semen quality as proven by the continuously decreasing sperm counts found by Levine et al. [6], research on male infertility has notably flourished in the last decades. Nonetheless, semen analysis remains the cornerstone of the initial evaluation in cases of male infertility. The World Health Organization has been publishing manuals since 1980, with the latest edition released in 2010 (a new one is expected in the upcoming months) and recommends cut-off values for semen parameters dramatically evolving over the years [7]. Advancements in research have recently allowed testing sperm at home, thus providing potential solutions for men who cannot overcome the burden of providing semen specimens in a “medical” setting [8]. These affordable, home-based sperm testing systems use smartphone technology and are mainly based on antibody reactions and microfluidics, reaching an accuracy of 95% to 98% in determining sperm concentration, thus becoming valid tools for preliminary screening [9]. However, caution is warranted, as these methods do not evaluate morphology, pH or volume and can generate a false sense of security, potentially delaying medical evaluation. Additionally, the spermogramme, while remaining the centerpiece of investigations in male infertility, fails to provide information regarding all functions of sperm; nor is it accurate in predicting the chances of success of assisted reproductive technology (ART) [10]. Therefore, great emphasis has been placed on novel tests that evaluate sperm function and abnormalities, with particular attention paid to sperm DNA integrity. Sperm DNA fragmentation (SDF) testing measures the quality of sperm as a DNA package carrier and has resulted in strong associations with impaired fertilization, slow early embryo development, reduced implantation and repeated miscarriage.

Currently, the most commonly used essays for evaluating SDF include terminal deoxynucleotidyl transferase-mediated dUTP nick-end labeling, sperm chromatin structure assay and sperm chromatin dispersion [11]. Furthermore, given the steep correlation between SDF and reactive oxygen species (ROS), the concept of measuring seminal oxidative stress as a means of sperm functional assessment has emerged. Indeed, studies have demonstrated that the use of chemiluminescent or fluorescent techniques for the assessment of ROS in semen might have prognostic value in distinguishing fertility potential [12]. However, although seminal oxidative stress can be determined by various assays, they still need to be validated through randomized clinical trials and are to be considered experimental until after validation [9,13]. Through a similar viewpoint, the investigation of the differential expression of sperm proteins by using highly specialized techniques, such as proteomics, may help in understanding the molecular pathways implicated in male infertility. The sperm proteome consists of a total of 6198 proteins, while 2064 proteins were reported in seminal plasma, and the expanding field of proteomics might identify useful biomarkers among these proteins for diagnosis and therapeutics in male infertility in the future [14]. 

Last but not least, the worldwide spread of the SARS-CoV 2 virus during the early days of 2020 induced severe global distress impacting hundreds of millions of lives worldwide. The impact of COVID-19 on fertility was initially devastating, as it resulted in the overall interruption of treatment that was resumed later on, after some progress in understanding the disease and the development of an effective vaccine. Inevitably, a plethora of research has been inspired regarding COVID-19 and fertility. However, the majority of studies examining the correlation between SARS-CoV-2 and male reproduction was observational, undersized and reported rather heterogeneous outcomes and, as such, do not provide definitive answers but rather suggestions to be considered cautiously. To date, there are no records of sexual transmission of SARSCoV2, while evidence of its presence in semen remains limited. For instance, six studies have investigated semen samples from infected patients, and the virus was detected in only 6 of 120 patients, all reported in a study by Li et al. [15,16]. Additionally, damage that is thought to be related to the virus was found in the testicle samples of men that died from COVID-19, as well as in histopathological samples in recovering men. It appears that the entrance of the virus into the testis cells is mediated through angiotensin-converting enzyme-2 (ACE2), as it also occurs in other tissues. DNA fragmentation, ROS formation, autoantibody production and ACE2-mediated effects might all play a role in the cellular damage. Furthermore, there has been evidence of significantly lower testosterone levels and sperm quality, as well as demonstrated impairment of spermatogenesis, as observed by Li et al. among 29 men (6 deceased and 23 recovering from COVID-19), thought to be partially related to an elevated immune response in testis [17,18]. 

Finally, there are two mRNA vaccines, BNT162b2 (Pfizer-BioNTech) and mRNA-1273 (Moderna), that received Emergency Use Authorization from the US Food and Drug Administration. Failure to assess reproductive toxicity in the clinical trials while developing the vaccine was listed as one of the reasons for manifested vaccine hesitancy. Thus, the results of the recently published study by Gonzalez et al., which evaluated sperm parameters in 45 men before and after two doses of COVID-19 mRNA vaccines, finding no significant decreases in any sperm parameter, were well received [19]. 

The last decades have indisputably afforded better clarity in male factor infertility; however, the persistence of numerous unresolved issues urges for well designed, randomized clinical trials in order to elaborate doubts, elucidate diagnostic and prognostic limitations and offer more options for treatment. 

## Data Availability

Not applicable.
